# Obtaining a Reliable Diagnostic Biomarker for Diabetes Mellitus by Standardizing Salivary Glucose Measurements

**DOI:** 10.3390/biom12101335

**Published:** 2022-09-21

**Authors:** Yangyang Cui, Hankun Zhang, Song Wang, Junzhe Lu, Jinmei He, Lanlan Liu, Weiqiang Liu

**Affiliations:** 1 Tsinghua Shenzhen International Graduate School, Tsinghua University, Shenzhen 518055, China; 2 Department of Mechanical Engineering, Tsinghua University, Beijing 100084, China; 3 Biomechanics and Biotechnology Laboratory, Research Institute of Tsinghua University in Shenzhen, Shenzhen 518057, China

**Keywords:** saliva, salivary glucose, standardizing, diabetes mellitus, sample collection

## Abstract

Salivary glucose is frequently utilized in diabetes mellitus (DM), and it might be proposed as a potential biomarker candidate for DM, as it is non-invasive and cost-effective and achieves adequate diagnostic performance for DM patients. However, salivary glucose levels may change under specific conditions. It is consequently essential to maintain a consistent strategy for measuring saliva, taking into account the possibility of external factors influencing salivary glucose levels. In this study, we analyzed salivary glucose levels under different handling conditions and donor-dependent factors, including age, interdiurnal variations, and collection and processing methods. A structured questionnaire was used to determine the symptoms and predisposing factors of DM. The glucose oxidase peroxidase method was used to estimate glucose levels in the blood and saliva of people in a fasting state. The aim of this study is to investigate the effect of such conditions on salivary glucose levels. We found that these extraneous variables should be taken into account in the future when salivary glucose is used as a predictive biomarker for DM.

## 1. Introduction

Diabetes mellitus (DM) is one of the most significant threats to public health in the twenty-first century [1]. The World Health Organization estimates that more than four million adults will die from DM and its complications in 2019, corresponding to one death every eight seconds [2]. Approximately 537 million people had DM in 2021. The total number of DM sufferers is expected to increase to 643 million by 2030 and to 783 million by 2045 [3,4]. 

However, the cause and etiology of DM are still unknown [5]. There are no treatments that can arrest the progression of DM, but there are ways to treat its symptoms. Managing the symptoms of this disease can lessen patient suffering and enhance their quality of life [6]. Furthermore, continuous monitoring can prevent or mitigate the difficulties observed during critical times. Sim et al. [7] highlighted that regular monitoring of patients’ blood glucose levels is of the utmost importance in order to ensure that they have a firm grasp on their situation and to prevent the onset of problems. Unfortunately, traditional blood glucose monitoring methods usually require blood collection, and the invasive monitoring process is associated with pain and inconvenience for patients [8]. To a certain extent, blood collection may cause mental stress in patients and affect their quality of life, especially for those who require blood glucose monitoring multiple times a day [9]. Therefore, noninvasive detection of blood glucose is a pressing issue with respect to the prevention, treatment, and management of DM that must be resolved immediately.

In their hunt for potential robust biomarkers for DM, scientists around the world have unanimously agreed to adopt standardized criteria. These requirements include samples that are simple to collect, have high sensitivity and specificity, are inexpensive in commercial test format, have defined cutoff values, and produce repeatable results over time [10,11]. We recently revealed the potential of salivary glucose as a prospective biomarker for DM, as it fits the aforementioned requirements. Our findings indicate that alterations in salivary glucose levels are unique to DM. This possible biomarker exhibited accurate parameters with extremely high sensitivity and specificity, in addition to being noninvasive and cost-effective [12,13].

Saliva collection is non-invasive, convenient, and simple to obtain; patients can collect samples themselves with minimal training. Saliva is composed of between 95% and 99% water, with 1–5% contents of several local and systemic components, including proteins, nucleic acids, glucose, electrolytes, and lipids [14,15]. These components interact and are responsible for the diverse functions of saliva, which reflect the health of the body [16]. Although saliva is an intriguing source for biomarker-based testing for a number of disorders, it is crucial to examine factors that can change salivary glucose levels, as saliva composition can change under specific conditions, such as sample procedures, processing methods, storage conditions, and many environmental and lifestyle variables [17,18]. Prior to the implementation of salivary glucose as a diagnostic biomarker for DM in future clinical practice, essential considerations must be discussed in order to confirm the notion that salivary glucose is an appropriate diagnostic biomarker for DM. The collection, handling, storage, and pathological variables of saliva samples are crucial for preventing variations in salivary glucose levels under varied conditions.

Saliva composition may vary depending on the time of day collected; consequently, research should be conducted to minimize the potential diurnal fluctuation in salivary glucose production [19]. Therefore, one important factor to consider is the time of saliva collection, as it can impact salivary glucose levels. Another important factor to consider is that most saliva collection devices on the market allow patients to collect stimulated saliva by stimulating the sample with various chemicals [20,21]. When a person is at rest, the submandibular gland is responsible for the majority of saliva production [22]. The parotid gland and the sublingual gland are only responsible for producing about 20% and 8% of saliva, respectively; however, when the production of saliva is stimulated, for example, by sodium chloride or by acid stimulation, the majority of generated saliva originates predominantly from the parotid gland [23]. Therefore, whether to stimulate, as well as stimulating substances and the degree of stimulation, should also be considered. Furthermore, the symptoms and predisposing factors of DM are also important factors affecting the level of salivary glucose [24,25].

Importantly, carbohydrates in stimulated and unstimulated saliva may vary due to underlying pathological conditions and/or exposure to medications and/or other chemicals or solutions, all of which may impede the correct derivation of data [26]. Therefore, biomolecules present in saliva in a healthy physiological state were selected, rather than those in a pathological state, so that saliva becomes the sample of choice for diagnostic and therapeutic purposes. The aim of this study is to investigate various procedures, collection methods (whether to rinse, chewing times, and stimulus conditions), pretreatment procedures (including storage conditions and freeze/thaw cycles), and conditioning extrinsic influences of factors on healthy participants (circadian rhythm, pathological conditions, symptoms, and predisposing factors) that may affect salivary glucose levels. Therefore, it is important to standardize salivary glucose collection and storage procedures, validate salivary glucose analytical techniques, and establish reference ranges for routine clinical use.

## 2. Materials and Methods

### 2.1. Ethics Statement

Saliva and blood were obtained from participants with valid informed consent. Tsinghua University’s local ethics commission authorized the collection of saliva and blood samples from humans (ethical approval code: Tsinghua.2021.74).

### 2.2. Participants

This study involved a total of 80 participants, including 40 DM patients. Inclusion criteria: patients diagnosed with diabetes; no history of diabetes complications, immune diseases, radiotherapy or chemotherapy; good oral hygiene; and disease duration of more than one year. Exclusion criteria: patients with large blood sugar fluctuations, those unable to communicate normally, patients with oral tumors, etc. An additional 40 healthy controls were included, with no significant difference in age or gender composition between the patient group and the control group and no evidence of systemic disease. Inclusion criteria for the control group: participants without diabetes, immunometabolic diseases, and other systemic diseases. Exclusion criteria: suffering from other immune or genetic diseases. On the day of data collection, all participants were free of fever and had excellent oral hygiene. In the event that an oral examination revealed poor oral hygiene, hyposalivation, oral complaints, or other oral problems (e.g., mucosal lesions or clinical signs of persistent periodontal disease), participants were promptly excluded from the research. The saliva of 40 healthy participants was used to examine the effect of saliva collection methods and pretreatment procedures on salivary glucose, whereas the saliva and blood of all 80 participants were utilized to examine the effect of pathological states on salivary glucose. A questionnaire was administered that covered 20 risk factors, including 10 symptom factors and 10 predisposing factors. Symptom factors included polydipsia, irritability, delayed healing, polyuria, decreased vision, polyphagia, obesity, weight loss, skin infection, and fatigue. Predisposing factors included gender, family history, number of pregnancies, age, location, exercise, drinking, smoking, occupation, and lifestyle.

### 2.3. Sample Collection

Participants were instructed to refrain from smoking, brushing their teeth, and eating or drinking 30 min before sample collection. Before samples were collected, the oral cavity was washed with water to eliminate food particles [27]. Standardized tubes with two sections and cotton were used to collect saliva. Cotton and dual-compartment tubes were manufactured by the same company (Salivette^TM^, Shanghai, China). The upper portion of the cotton-filled test tube features a hole; following centrifugation, the saliva was recovered in the lower portion to be utilized for analysis. In the same clinical room, all saliva samples were collected in order. To prevent the degradation of sensitive peptides, all samples were collected in prechilled polypropylene tubes on ice. Finally, samples were transported to the laboratory and centrifuged at −20 °C for future use. The saliva collection process is consistent with that used in our previous study [28,29]. In this study, the whole saliva was collected. All sampling processes comply with safety regulations [30].

We investigated different procedures, collection methods (whether to rinse, chewing times, and stimulus conditions), pretreatment procedures (including different storage conditions and freeze/thaw cycles), and conditioning extrinsic influence of factors through participants (circadian rhythm, pathological conditions, symptoms, and predisposing factors) that may affect salivary glucose levels. Therefore, it was important to standardize salivary glucose collection and storage procedures, validate salivary glucose analytical techniques, and establish reference ranges for routine clinical use. The same 40 healthy participants were selected for each of the different conditions. The particular experimental procedures are as follows:

#### 2.3.1. Procedures of Whether to Rinse

In this part, 40 healthy participants were selected, and three collection methods were proposed: chewing a cotton swab 70 times in 1 min, chewing 70 times in 3 min, and containing a cotton swab between the teeth for 5 min. The participants did not brush their teeth in the fasting state and collected saliva with the three methods after rinsing their mouths. The next day, the participants were instructed to collect saliva according to the above three methods again 30 min after brushing their teeth in the fasted state in the morning.

#### 2.3.2. Procedures of Chewing Times

In this part, 40 healthy participants were selected. After morning tooth brushing in the fasting state, the oral cavity was kept irritant-free for at least 30 min, and participants chewed cotton swabs 40–50 times per minute, 50–60 times per minute, or 60–70 times per minute to collect saliva.

#### 2.3.3. Procedures of Centrifugation Speed

In this part, 40 healthy participants were selected. After morning tooth brushing in the fasting state, the oral cavity was kept irritant-free for at least 30 min; then, saliva was collected with cotton and transferred from the cotton to the tube by centrifugation using different centrifugation speeds. The centrifugation speeds included 2000 r/min, 3000 r/min, 4000 r/min, and 5000 r/min. Finally, the collected saliva was identified and evaluated.

#### 2.3.4. Procedures of Stimulus Conditions

In this part, 40 healthy participants were selected to stimulate the oral cavity with 0.075 M, 0.15 M, or 0.30 M sodium chloride and 0.005, 0.01 M, or 0.02 M citric acid, using water as the baseline. On six different days, each participant completed six one-hour sessions. At room temperature, saliva was collected after stimulation with water, each of the three levels of sodium chloride citric acid, and each of the three levels of one of the flavor compounds. A water stimulus was administered at the start (baseline) and end (check for return to baseline) of the session. For each stimulus, 5 mL sample was delivered. The participant inserted the full sample into their mouth, gently swirled it about for 15 s, expectorated, waited 45 s, and then repeated the procedure for the remaining four samples. Saliva was collected throughout the five-minute stimulation period. Before the following stimulus, the participant washed his or her mouth three times with deionized, distilled water. The order of stimulus presentation was the same for all individuals and remained unchanged during the six different sessions over the course of the six experimental days

#### 2.3.5. Procedures of Storage Conditions

In this part, 40 healthy participants were selected. Half of the collected saliva samples were placed in a centrifuge tube, with storage methods including immediate detection, detection after 2 h at room temperature, and 2 h at 4 °C.

#### 2.3.6. Procedures of Freeze/Thaw Cycles

In this part, 40 healthy participants were selected. Half of the collected saliva samples were divided into 2 equal parts for storage. Then, one of the two parts was divided into nine small equal parts. The parts were used for the procedure described in Section 2.3.5. The remaining saliva samples were stored in a freezer. The saliva samples were stored for 5 d, 10 d, 15 d, 20 d, 25 d, or 35 d, then taken out and centrifuged, and the supernatant was taken for testing. The collected saliva was tested for glucose to determine the freezing time. Furthermore, a large portion of unused saliva was tested synchronously, and the effect of freezing cycles on the glucose level in saliva was analyzed.

#### 2.3.7. Procedures for Collection Time

In this part, 40 healthy participants were selected, with saliva samples collected at the same six time periods. The collection time was divided according to three meals. Saliva was collected before and after the three meals. All participants ate the same food. “After meal” refers to 2 h after the meal. Collection times were 7:30–8:00 (fasting blood glucose, before breakfast), 9:30–10:00 (after breakfast), 11:30–12:00 (before lunch), 13:30–14:00 (after lunch), 15:30–16:00 (before dinner), and 17:30–18:00 (after dinner). All saliva samples were collected in a clinical room at the same time.

#### 2.3.8. Procedures of Pathological Factors

In this part, 40 healthy controls and 40 DM patients were selected to collect salivary and blood glucose in different time periods to compare the salivary glucose levels and blood glucose levels at different time periods, and the effects of pathological factors on salivary glucose were analyzed. The time periods were the same as the collection times in listed in Section 2.3.7 (six time periods: 8:00, 10:00, 12:00, 14:00, 16:00, and 18:00).

### 2.4. Estimation of Blood and Salivary Glucose

Blood and salivary glucose levels were determined using the glucose oxidase peroxidase (GOD-POD) method. Blood and saliva samples were separated into three test tubes and labeled “Blank”, “Standard”, and “Test”. The following steps were implemented: first, add 50 μL of different concentrations of standards to the “Standard; add 40 μL of sample diluent to the wells of the samples to be tested, and then add 10 μL of the samples to be tested; add 100 μL of enzyme-labeled reagents to each well, except for the “Blank”; seal the plate with a sealing film and incubate at 37 °C for 60 min; dilute the 20-fold concentrated washing solution with 20-fold distilled water for later use; remove the sealing membrane, discard the liquid, spin dry, fill each well with washing solution, let set aside for 30 s and discard, repeat 5 times, and pat dry; add 50 μL of color developer A to each well, then add 50 μL of color developer B, gently shake and mix, and develop color at 37 °C for 15 min in the dark; stop adding to each well; terminate the reaction was by adding 50 μL of solution; finally, set the “Blank” to zero, and measure the absorbance (OD value) of each well in sequence at a wavelength of 450 nm with a microplate reader (Bio-Rad iMark, 168-1130, Tokyo, Japan).

### 2.5. Statistics

The data were analyzed using Graphpad 8.0 (GraphPad Software, San Diego, CA, USA). The means and standard deviations (SDs) of each group were computed. ANOVA with Student’s independent t-test was used to assess between-group variation, and Spearman’s coefficient was used to measure the association between variables. Significance was set at *p* < 0.05, whereas *p* < 0.01 was regarded as highly significant.

## 3. Results

### 3.1. Questionnaire Survey

**Figure 1:**
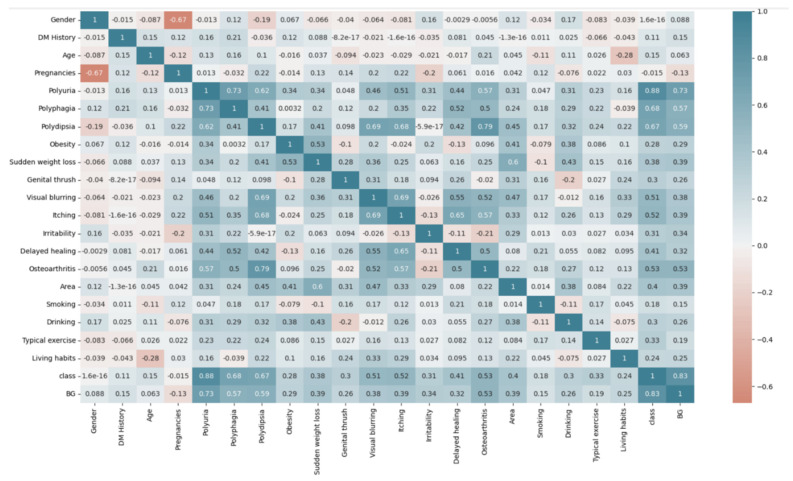
Questionnaire risk factor correlation.

Analysis revealed minimal correlation between risk factors. If all the risk factors are combined to predict the blood glucose level, a better effect will be achieved. A total of 80 participants were included in the questionnaire, aged 22 to 60 years old, as shown in Figure 1. We found that polyphagia, polydipsia, polyuria, and significant weight loss occurred only in DM patients, indicating that these are symptoms of DM, although most DM patients do not have the typical symptoms of “three more and one less”. Second, the feeling that the eyes are easily fatigued, as well as gradually declining, and blurred vision, also increases the risk of DM patients. Finally, age, DM history, and number of pregnancies were also found to be strongly associated with DM.

### 3.2. Whether to Rinse

In this part, 40 healthy participants were selected. Three following collection methods were proposed: chewing a cotton swab 70 times in 1 min, chewing 70 times in 3 min, and containing a cotton swab between the teeth for 5 min. On the second day, the participants were instructed to collect saliva again according to the three methods mentioned above 30 min after brushing their teeth on an empty stomach in the morning. The results are shown in Table 1. The results show that independent of the chewing method, there was no significant difference in salivary glucose levels between 30 min after fasting without brushing and rinsing and 30 min after fasting with brushing and rinsing (*p* > 0.05).

### 3.3. Chewing Times

The results show that there was no statistical difference in salivary glucose and saliva volume between 40–50 times per minute, 50–60 times per minute, and 60–70 times per minute, as shown in Table 2. There was no gender difference in salivary glucose level or saliva volume under different chewing times (both *p* > 0.05). Therefore, considering that participants can easily complete the number of chews and obtain a stable amount of saliva, the chewing condition of 40–50 times per minute is recommended.

### 3.4. Centrifugation Speed

In this study, we found that different processing methods of salivary glucose samples, such as different centrifugation speeds, have an impact on the detection results of salivary glucose levels. In the process of processing salivary glucose samples, with increased centrifugation speed, salivary glucose levels first increased; then, after reaching a threshold speed, salivary glucose levels remained unchanged, as shown in Table 3. The overall mean saliva glucose level change was statistically significant at *p* < 0.05 when the centrifugation speed was less than 3000 r/min.

### 3.5. Stimulus Conditions

After oral stimulation with water as a baseline, aqueous solutions of sodium chloride (0.075, 0.15, and 0.30 M) and citric acid (0.005, 0.01, and 0.02 M) were used to collect unilateral parotid saliva from 40 participants. The amount of saliva produced increased in proportion to the intensities of each taste stimulation. The maximum flow rate was elicited by citric acid at levels that were otherwise equivalent in terms of the intensity of the taste.

Table 4 presents uncorrected data for samples of unstimulated and stimulated saliva. A considerable increase in mean flow rate accompanied the commencement of stimulation. The flow rate then appeared to diminish as stimulation continued. The overall mean flow rate change was statistically significant at *p* > 0.05, but the difference between the first and second stimulated samples was not statistically significant. The mean level of total salivary glucose decreased over the period of stimulation. All mean level differences were statistically significant. In addition, there was no significant change between the first and second samples that were stimulated.

### 3.6. Storage Conditions

In this part, saliva samples from 40 healthy participants were selected, and each case was divided into nine equal parts. The enzyme method was used for immediate detection, detection after storage at room temperature for 2 h, and detection after storage at 4 °C for 2 h. As shown in Table 5, there was no significant difference in the salivary glucose level after 2 h at room temperature and 2 h at 4 °C (both *p* > 0.05). The saliva samples were stored under different conditions depending on the follow-up study. In this part, the saliva samples were stored in aliquots at −20 °C for 35 days. We found that storage conditions had no considerable effect on the salivary glucose level, indicating the stability of salivary glucose. Salivary glucose can be store for at least one month at −20 °C.

### 3.7. Freeze/Thaw Cycles

To maintain the long-term stability of salivary glucose, we recommend freezing saliva samples in aliquots after collection at −20 °C. Small aliquot amounts are the optimal solution to avoid freeze/thaw cycles so that one aliquot can be thawed at a time. Samples were collected and stored at 4 °C for further measurement of salivary glucose levels. There was no difference in salivary glucose levels between samples that went through none or only two freeze/thaw cycles. The salivary glucose level gradually fell after more than two freeze/thaw cycles, notably lower than after two cycles, as indicated in Table 6. This drop in salivary glucose level could be attributed to denaturation of other compounds in the saliva (*p* < 0.01), which affects the salivary glucose level. In biological samples, freezing and thawing methods can cause level gradients. To avoid such gradients, the levels in the tube must be properly mixed.

### 3.8. Collection Time

**Figure 2:**
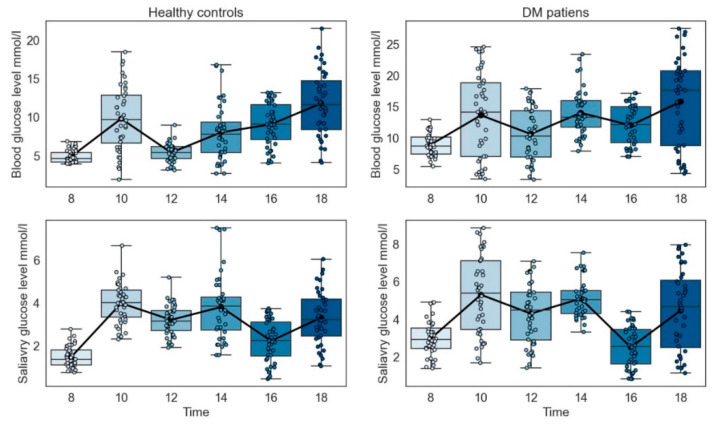
Changes in salivary glucose levels of participants at different collection times.

Saliva from 80 participants was collected for six time periods, and blood glucose levels were measured for each time period. We found that blood glucose and salivary glucose levels were the lowest and most stable on an empty stomach, as shown in Figure 2. We also found that under physiological conditions, a large amount of glucose was absorbed into the blood after eating, resulting in a temporarily increased blood glucose level before returning to the original level after 2 h. The trend of salivary glucose is the same as that of blood glucose, with a rapid postprandial increase in salivary glucose, followed by a gradual decrease over time. The overall mean saliva glucose level change over time was statistically significant at *p* < 0.05.

### 3.9. Pathological Effects

**Figure 3:**
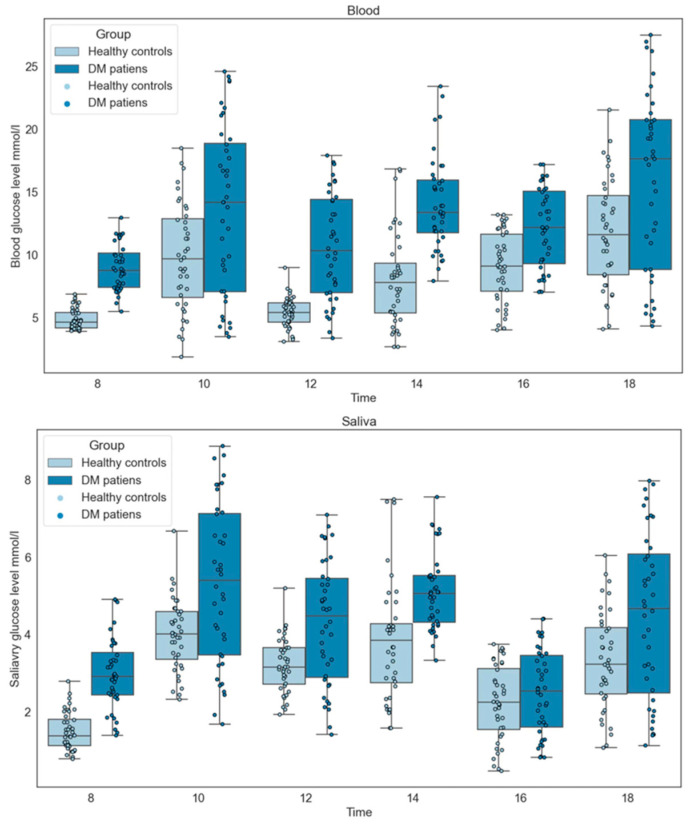
Comparison of saliva and blood glucose levels between DM patients and healthy controls.

A total of 40 healthy controls and 40 DM patients were selected and gender-matched. We found that the salivary glucose and blood glucose levels of DM patients were higher than those of the control group, as shown in Figure 3. Our results show that there are large individual differences in salivary glucose and blood glucose among both healthy and DM patients. Our selection criteria for participants were obvious, so the questionnaire aspect of this study was necessary.

## 4. Discussion

The results of this study show that the efficacy of salivary glucose as a diagnostic marker for DM is dependent on standardized salivary preanalysis, collection, and processing methodologies. In the current study, we found that standardized saliva collection and processing techniques are key to minimizing interindividual variability in saliva composition.

By comparing salivary glucose levels under different collection conditions, chewing times, centrifugation speeds, stimulus conditions, storage conditions, freeze/thaw cycles, and collection times, we concluded that brushing before collection did not affect salivary glucose levels. Saliva was collected after participants chewed a cotton swab 40–50 times in 1 min, and the obtained saliva volume and salivary glucose level remained stable. The centrifugation speed of 3000 r/min indicates that the salivary glucose level did not increase. The stimulated salivary flow rate increased, but with increased stimulation, both the salivary flow rate and salivary glucose level decreased. Saliva samples can be stored at room temperature or 4 °C for a short period of time or at −20 °C for at least one month. We also found that salivary glucose levels decreased after two freezing cycles, so storing in smaller portions is recommended. Furthermore, salivary glucose levels are considerably affected by day and night, with the fasting salivary glucose level found to be the most stable. Finally, fasting saliva was the main research object in the current research. As stated in detail in our previous study, there is a strong positive association between enzymatically determined blood and salivary glucose in DM patients, and salivary glucose levels can differ considerably among DM patients [12,13].

To date, studies have been conducted on the detection of DM through saliva, but there are considerable differences in terms of methods of saliva collection. Dhanya et al. [31] conducted a study on the detection of DM by salivary glucose, asking participants to rinse their mouths with distilled water and keep their mouths irritated for 5 min prior to saliva collection. During collection, participants bowed their heads without swallowing for 5 min. The saliva produced in the oral cavity was collected into a common collection tube, and the remaining saliva in the oral cavity was spat into the collection tube after 5 min. In another study to explore the changes of salivary glucose, amylase, immunoglobulin, and other components in DM patients, the above method was also used to collect data [32,33]. Because the direct saliva collection method may result in oral debris mixed in the saliva and the collection process makes the participants feel uncomfortable, researchers are increasingly using a special Salivette saliva collection tube for saliva collection, which contains cotton, as it is more hygienic and convenient to absorb saliva through a cotton swab. In a study of salivary cortisol, Uygun et al. [34] instructed participants to soak a cotton swab in a saliva collection tube for 2 to 3 min. Mészáros et al. [35] collected saliva by having participants sublingually swallow a cotton swab for 1 to 2 min. Ciurli et al. [36] instructed participants to chew a cotton swab in a Salivette saliva collection tube for 1 min. In this study, saliva was collected using a Salivette saliva collection tube. It is recommended to chew a cotton swab 40–50 times per minute. This is positively clinical operable, with a short collection time, stable saliva volume, and easy acceptance by patients.

A number of studies have also explored the collection of stimulated and unstimulated salivary glucose samples when examining collection methods, and our results confirm that glucose levels are lower in stimulated saliva than in unstimulated saliva [37,38]. Dhanya et al. [31] found that the salivary glucose level collected under unstimulated settings is higher than that collected under stimulated conditions, which is compatible with the findings of this study. Other investigations have demonstrated that the salivary glucose level collected under unstimulated and stimulated settings does not significantly differ [39]. Because individuals may not readily accept stimulation and stimulated saliva contains more water than unstimulated saliva, unstimulated saliva may be a better indicator of normal physiological condition. Takeda et al. [40] investigated the chemical level of the saliva of healthy subjects under various conditions and found that the level of nearly all metabolites in unstimulated saliva was higher than in stimulated saliva. Jha et al. [41] also discovered that compared to stimulated saliva, average salivary glucose levels in stimulated saliva were higher in both control and non-control DM patients. This also validates the conclusions drawn in this study. Furthermore, our study extends knowledge on the effects of different stimulation types and degrees of stimulation on salivary glucose levels. According to research presented by Newbrun [42], the abundant salivation induced by citric acid is a dilution mechanism for safeguarding the oral mucosa. As reported by Funakoshi [43], salivation in reaction with sodium chloride showed a considerably steadier increase. The induction of flow by sodium chloride could alternatively be interpreted as a biologically protective response to an abrupt increase in the number of ions in the mouth. The curvilinear flow response to citric acid suggests that the oral cavity has a maximum capacity for fluid secretion, and the high flow rates produced by citric acid stimulation may cause the flow rate to approach this maximum, resulting in a leveling off of flow. We also found that salivary glucose levels continued to decrease with increased stimulation levels, whereas salivary flow increased first and then decreased.

Reported centrifugation speeds and storage methods following saliva collection also vary considerably between studies, and the majority of clinical chemistry tests in blood or saliva samples require centrifugation prior to analysis to separate blood cells and other components, such as glucose [44]. Although this preanalytical operation is performed on a daily basis in many medical laboratories around the world, the impact of centrifugation on test results is not often reported. The separation efficiency of the centrifugation process is mainly determined by the centrifugation speed, so in this study, we explored the effects of different centrifugation speeds on salivary glucose levels. We found that salivary glucose levels increased with increased centrifugation speed but that beyond a threshold centrifugation speed, the salivary glucose level remained unchanged, possibly due to insufficient centrifugation and residual saliva in the cotton swab with a low centrifugation speed. However, when the centrifugation speed reaches a sufficient threshold value, the salivary glucose level remains unchanged.

Numerous experts have also undertaken research on various storage techniques. Mészáros et al. [35] stored salivary cortisol samples at −20 °C after collection prior to testing. In another study, saliva samples were stored in a 4 °C refrigerator after collection, with salivary cortisol detected within 24 h [45]. Saliva cannot be tested immediately after collection and requires short-term transportation, so we compared instant detection at 4 °C and following 2 h of storage at room temperature. We observed no significant difference with short-term storage, which provides for the transportation of saliva. Although transporting biological samples between laboratories at −20 °C may make it easier to preserve samples, such conditions may have a negative impact on salivary glucose-related outcomes. Salivary glucose levels decreased somewhat but not significantly in aliquots thawed once and stored at −20 °C for 35 days compared to reference samples (aliquots stored at −20 °C until thawed for analysis). Regardless, our findings suggest that salivary glucose levels remain unchanged for at least 35 days in saliva samples stored at −20 °C; however, avoiding storage at −20 °C beyond this time frame is recommended. We also demonstrated that it is preferrable to send samples for subsequent analysis as soon as possible. If samples cannot be analyzed immediately, they can be temporarily stored at −20 °C for one month.

Whereas freezing cycles have an influence on samples, freezing and thawing techniques may generate level gradients in biological samples [46]. To prevent such gradients, the levels in the tube must be properly mixed. Several studies have examined the biophysical properties of the contained materials. In these studies, it was demonstrated that repeated freeze–thaw cycles may affect the stability and level of substances in saliva [47,48]. This phenomenon was verified in our study, and glucose levels were significantly reduced after the third repeated freeze–thaw cycle, which is consistent with the results of previous studies. This highlights the importance of avoiding excessive freeze–thaw cycles to minimize sample degradation, which can lead to misleading salivary glucose levels.

In summary, samples can be subjected to short-term or long-term storage, depending on the purpose of the study. Furthermore, factors such as the size of the sample, the difficulty of collection and processing, logistics, and sample library management should be comprehensively considered, and it is necessary to determine whether to store samples locally or centrally. If samples are to be stored for more than 35 days, centralized storage is recommended, and we recommend using two storage locations located far apart with different power supply systems to mitigate the effects of equipment failure or natural disasters. In addition, each storage location should have at least one empty spare refrigerator in case of instrument failure. Furthermore, all samples should not be packaged at the time, samples should be stored separately, and repeated freezing and thawing should be avoided during the storage process.

The relationship between salivary glucose levels and circadian rhythms was also explored in the present study. Salivary composition may vary depending on the time of day of collection [19,49]. Our findings demonstrate that salivary glucose levels fluctuate throughout the day. Although several studies have suggested that DM-releasing and metabolic pathways may respond promptly to changes in the circadian clock [50], our findings are consistent with the discovery that salivary glucose levels fluctuate over time, indicating a considerable circadian rhythmic influence. We found that both blood glucose and salivary glucose values were lowest when fasting. The physiological activities of adults show a certain biological rhythm, and some physiological activities undergo periodic changes in diurnal cycles when the activities of the brain and various organs of the body are at their lowest point [51]. Most people are in a sleep state at night, and sympathetic nerve excitability is low, the inhibitory effect on insulin is reduced, the levels of glucagon and adrenocortical hormone are the lowest, as is glycogen decomposition. Both gluconeogenesis and gluconeogenesis are reduced at night, blood glucose values are low, and the probability of hypoglycemia is high [52]. Fasting blood glucose and salivary glucose are the most stable at night. After 8:00 am is often the time when people start getting up and moving. Changes in the environment affect the excitability of autonomic nerves, most of which show increased sympathetic nerve excitability, resulting in increased blood glucose [53]. Furthermore, under physiological conditions, a large amount of glucose is absorbed into the blood after eating, and the blood glucose level temporarily increases before returning to the original level after 2 h. Blood glucose levels are at a constant fasting level between 7:00–8:00 the next morning. Therefore, a fasting blood collection time of 7:00–8:00 is recommended to obtain clinical blood glucose values that best reflects the physiological condition.

In this study, salivary glucose levels in DM patients and healthy controls were also analyzed. The healthy controls had lower mean salivary glucose levels than the DM patients. Similar to our findings, Mahdavi et al. [54] and numerous other authors have reported the presence of salivary glucose in DM patients. Ivanovsi et al. [55] observed that DM patients had higher salivary glucose levels than healthy controls. In a study conducted by Abikshyeet et al. [56], a positive, statistically significant association was discovered between saliva and blood glucose in DM patients and healthy controls. Therefore, salivary glucose can be utilized as an indicator of the level of glucose in DM patients’ blood. Our findings align with the results of a study by Arakawa et al. [57], suggesting that both DM patients and healthy controls have detectable glucose in their saliva and that DM patients have higher salivary glucose levels than healthy controls. This is likely due to the fact that glucose is a tiny molecule that can easily travel through the vascular membrane fluid in the gums and combine with saliva [58]. In DM patients, the end products of blood glucose metabolism cause microvascular and vascular damage to the basement membrane of salivary gland cells, resulting in an increase in salivary glucose levels [59]. In addition, Brown University researchers developed a new biochip sensor that can selectively measure glucose levels in human saliva, concluding that standardizing participant selection criteria and saliva collection methods are crucial steps, allowing diabetics to test their glucose levels without drawing blood [60,61]. In this study, we standardized saliva collection methods. Participant selection criteria were determined by means of questionnaires. The questionnaires contained elicited possible influences, including DM symptom factors, which may affect salivary glucose levels.

Polyuria, polyphagia, polydipsia, abrupt weight loss, hazy eyesight, etc., are typical DM symptoms [62,63]. Recent research by Harris et al. [64] indicates that DM may be present 7 or even 12 years prior to clinical diagnosis. During this period, deadly complications, such as heart disease, foot ulcers, kidney damage, and other forms of multiple organ damage, develop progressively in patients [65]. If DM is diagnosed and treated promptly, these consequences can typically be managed. With the advancement of computer technology, diseases can be identified more precisely, saving time and money. To predict diseases using data mining, disease symptoms and clinical data are required [66], which was the function of the questionnaire in the present investigation. These levels affect salivary glucose levels. In the questionnaire survey, we found that when eating more, drinking more, urinating more, weight loss are obvious symptoms of DM, although the typical symptoms of “three more and one less” were not observed. Most DM patients do not have the typical symptoms of “three more and one less”, which can easily lead to DM patients neglecting blood glucose health. Furthermore, participants with DM were found to be at an increased risk of experiencing eye fatigue, progressive vision loss, and blurred vision.

All of these factors identified through the questionnaire are crucial considerations that must be explored and investigated in future multicenter studies. As indicated by the intriguing and compelling evidence provided obtained in the present study, variability in salivary glucose is influenced by external influences. In addition, this study provides evidence that salivary glucose is a sensitive and noninvasive indicator of DM. Before collecting saliva samples, the participant selection criteria must be strictly controlled according to experimental needs. Interested participants should be carefully selected based on questionnaire responses, detailed history, and a complete clinical examination, if applicable, in order to considerably reduce sampling error and improve the accuracy of non-invasive detection of salivary glucose instead of blood glucose. 

This study is subject to some limitations. First, our result only prove that storing saliva samples at −20 °C for one month did not influence salivary glucose levels; it remains to be determined whether saliva samples can be stored at −80 °C for longer durations. In addition, the sample size of this study is modest, so it is important to increase the sample size in order to confirm the consistency of salivary glucose levels in association with various detection methods and matrices in future studies. In addition, although we standardized a saliva collection method and optimized participant selection criteria in this study, precautions with respect to salivary collection were limited, and many factors affect saliva secretion besides the factors addressed in the questionnaire, such as Sjögren’s syndrome, periodontitis, etc. These variables can be controlled to determine whether these factors have an impact on salivary glucose levels. Finally, it is well-known that saliva is a complex matrix that contains other biomolecules that could interfere with analysis. Therefore, the effects of other substances in saliva on salivary glucose should also be considered in future research.

## 5. Conclusions

A lack of standardized preanalytical procedures in biomarker studies can lead to considerable differences in the obtained results. Therefore, in this study, we explored different collection methods (including whether to rinse, chewing times, and stimulus conditions), preprocessing procedures (including storage conditions and freeze/thaw cycles), and external influencing factors (circadian rhythms, pathological conditions, symptoms, and predisposing factors) to standardize salivary glucose collection and storage procedures, validate salivary glucose analytical techniques, and establish reference ranges for routine clinical use.

In this study, we discovered that salivary glucose levels are affected by external factors, such as stimulus conditions and repeated freeze/thaw cycles. However, storing samples at −20 °C for up to 35 days and for no more than three freeze/thaw cycles did not appreciably alter salivary glucose levels. Such conditions enable sample transfer and analysis, making the process more efficient and economical. Therefore, employing a uniform data collection and storage strategy is recommended to duplicate our study results and verify that preanalytical factors do not influence the results. Furthermore, we recommend freezing saliva samples into aliquots at –20 °C to ensure the long-term stability of salivary glucose.

We also discovered that salivary glucose is impacted by circadian rhythms; therefore, the time of saliva collection is crucial. In addition, saliva samples should be collected on an empty stomach at the same time of day to eliminate the possibility of fluctuating salivary glucose levels. Extrinsic factors, such as DM symptoms and predisposing factors, may influence the donor’s salivary glucose production. Although salivary glucose levels may fluctuate in response to these variables, these variations did not occur in the same patients. Therefore, in order to compare salivary glucose levels between healthy and DM patients, we suggest rigorous participant inclusion criteria, as the sampling of participants is essential to the experiment’s correctness.

All of these extrinsic factors must be considered when validating salivary glucose as a DM biomarker in the future. In conclusion, we recommend that all research groups and laboratories utilize the abovementioned techniques for sample processing and preservation, with a particular emphasis on verifying salivary glucose levels as a biomarker of DM. In addition, we recommend the examination of questionnaire variables associated with variations in salivary glucose levels. These recommendations provide a checklist for standardizing biomarker sample collection in order to obtain large quantities of well-characterized samples.

## Figures and Tables

**Table 1:** The effect of tooth brushing on salivary glucose. (Unit: mmol/L).

Whether to Rinse	No	Yes	*p*
70 times 1 min	1.63 ± 0.79	1.22 ± 0.29	0.28
70 times 3 min	0.86 ± 0.27	0.85 ± 0.11	0.946
5 min with cotton swab	1.67 ± 1.01	1.26 ± 0.74	0.288

**Table 2:** Effects of chewing times on salivary glucose. (Unit: mmol/L).

Measure	Chewing Times	Total	Female	Male	*p*
Salivary glucose	40–50 times/min	1.54 ± 0.51	1.87 ± 0.51	1.33 ± 0.38	0.087
	50–60 times/min	1.15 ± 0.34	1.03 ± 0.33	1.27 ± 0.38	0.409
	60–70 times/min	1.27 ± 0.41	1.18 ± 0.66	1.36 ± 0.37	0.601
Saliva volume	40–50 times/min	0.84 ± 0.14	0.76 ± 0.17	0.89 ± 0.03	0.174
	50–60 times/min	1.02 ± 0.43	1.09 ± 0.33	0.97 ± 0.62	0.691
	60–70 times/min	1.09 ± 0.40	1.35 ± 0.29	0.92 ± 0.32	0.097

**Table 3:** Effect of centrifugal speed on salivary glucose. (Unit: mmol/L).

Centrifugation Speed (3 min)					
1000 r/min	2000 r/min	3000 r/min	4000 r/min	5000 r/min	*p*
1.42 ± 0.17	1.50 ± 0.23	1.53 ± 0.53 *	1.53 ± 0.64	1.53 ± 0.66	0.026

* the maximum glucose level, which remained unchanged increased speed.

**Table 4:** Effects of different stimulation conditions on salivary glucose. (Unit: mmol/L).

		Stimulus Level *				
Measure	Stimulus	0	1	2	3	*p*
Salivary glucose	Sodium chloride	1.67 ± 0.23	1.63 ± 0.28	1.47 ± 0.21	1.41 ± 0.14	0.812
	Citric acid	1.63 ± 0.53	1.58 ± 0.49	1.49 ± 0.27	1.42 ± 0.15	0.079
Salivary flow rate	Sodium chloride	0.51 ± 0.11	1.77 ± 0.21	2.01 ± 0.23	1.53 ± 0.17	0.054
	Citric acid	0.72 ± 0.07	2.26 ± 0.31	2.38 ± 0.15	1.67 ± 0.14	0.068

* Sodium chloride: 0.075 M, 0.15 M, and 0.30 M; citric acid 0.005 M, 0.01 M, and 0.02 M.

**Table 5:** Effects of different storage conditions on salivary glucose. (Unit: mmol/L).

0 min *	Room Temperature ** 2 h	4 °C 2 h ***	5 d	10 d	15 d	20 d	25 d	30 d	*p*
1.53 ± 0.53	1.53 ± 0.49	1.53 ± 0.54	1.53 ± 0.61	1.53 ± 0.72	1.53 ± 0.53	1.51 ± 0.59	1.51 ± 0.76	1.51 ± 0.89	0.037

* 0 min indicates that the saliva sample was tested immediately after collection. ** Room temperature 2 h indicates that the saliva sample was stored for 2 h at room temperature before testing. *** 4 °C 2 h indicates that the saliva sample was stored for 2 h at 4 °C before testing.

**Table 6:** Effects of different storage conditions on salivary glucose. (Unit: mmol/L).

Freeze/Thaw Cycles							
0	1	2	3	4	5	6	*p*
1.53 ± 0.53	1.53 ± 0.61	1.53 ± 0.72	1.52 ± 0.53	1.51 ± 0.59	1.49 ± 0.76	1.48 ± 0.89	0.008

## Data Availability

Not applicable.
